# LncRNA SLCO4A1-AS1 facilitates growth and metastasis of colorectal cancer through β-catenin-dependent Wnt pathway

**DOI:** 10.1186/s13046-018-0896-y

**Published:** 2018-09-10

**Authors:** Jiangtao Yu, Zhiyang Han, Ziquan Sun, Yue Wang, Ming Zheng, Chunfang Song

**Affiliations:** 1 Department of General Surgery, the Sanya Hongsen Hospital of Harbin Medical University, Fenghuang Road, Sanya, 572000 China; 2 Department of General Surgery, the First Affiliated Hospital of Harbin Medical University, Harbin, 150001 China; 3 Department of Pharmacology and Toxicology, Wright State University, Fairborn, OH 45435 USA; 4 Department of Ultrasonography, the Sanya Hongsen Hospital of Harbin Medical University, Sanya, 572000 China

**Keywords:** SLCO4A1, AS1, Colorectal cancer, Tumorigenesis, β-catenin

## Abstract

**Background:**

Emerging evidence has shown long noncoding RNAs (lncRNAs) exert important roles in colorectal cancer (CRC) tumorigenesis. However, most lncRNAs involved in this process remain undefined and the underlying molecular mechanisms mediated by lncRNAs are largely unknown.

**Methods:**

An unbiased screening was used to identify novel lncRNAs involved in CRC according to an online-available data dataset. In situ hybridization (ISH) and qRT-PCR was used to detect lncRNA expression patterns. CCK8, colony formation, fluorescence activated cell sorter (FACS), transwell, xenograft nude mouse model and western blot assays were used to analyze the functions of SLCO4A1-AS1. RNA-pulldown, western blot, RNA fluorescence in situ hybridization (RNA-FISH) and electrophoretic mobility shift assay (EMSA) assays were utilized to explore the molecular mechanism of SLCO4A1-AS1.

**Results:**

LncRNA SLCO4A1-AS1 was significantly upregulated in CRC tissues and its overexpression was closely related with poor prognosis and tumor metastasis. By knocking down SLCO4A1-AS1, we found that SLCO4A1-AS1 promoted the proliferation, migration, invasion and epithelial–mesenchymal transition (EMT) of CRC cells in vitro, as well as inhibited cell apoptosis. Moreover, SLCO4A1-AS1 dramatically delayed tumor propagation in vivo. Mechanistically, SLCO4A1-AS1 activates Wnt/β-catenin signaling. SLCO4A1-AS1 enhanced the stability of β-catenin by impairing the interaction of β-catenin with GSKβ and inhibiting its phosphorylation. Finally, restoration of β-catenin protein level rescued the proliferation, migration and invasion in SLCO4A1-AS1-depleted CRC cells.

**Conclusion:**

SLCO4A1-AS1 serves as an oncogenic role in CRC through activating Wnt/β-catenin signaling pathway. And SLCO4A1-AS1 might be a useful biomarker for CRC diagnosis and prognosis.

## Background

As one of the most prevalent cancers, colorectal cancer (CRC) has become the third-leading cause of cancer-related death worldwide every year [1]. Hyperactivation of some signaling pathways, including wnt/β-catenin, PI3K/AKT, JAK/STAT signaling pathways and so on, often contributes to the development, progression, metastasis and resistance to chemotherapy of CRC [2–4]. Therefore, understanding the underlying molecular mechanisms that regulate these signaling pathways is urgent and essential for developing efficient target-specific drugs against CRC.

Long noncoding RNAs (lncRNA) are a class of transcripts of longer than 200 nucleotides and possess no protein-coding potential [5–7]. Emerging evidence shows that lncRNAs are critical regulators involved in various biological processes via multiple mechanisms [8, 9], such as development, immune regulation and especially tumorigenesis [10–12]. Importantly, accumulating studies have proven that aberrant expression of lncRNAs is closely related to various human cancers [13–15]. For example, lncRNA TUG1 interacts with miR-138-5p to enhance cervical cancer progression by upregulating SIRT1 expression [16]. LncRNA SNHG15 recruits EZH2 to inhibit P15 and KLF2 expression, and promote pancreatic cancer proliferation [17]. In addition, elevated expression of SNHG6 promotes gastric cancer cell proliferation and EMT, and correlates with poor prognosis. This trend has been observed and proven in diverse tumors including prostate cancer, breast cancer, CRC and so on [18–20]. Recently, many lncRNAs such as LINC01567, HOXB-AS3 and BANCR are reported to be involved in the occurrence and metastasis of CRC [21–23]. However, the underlying molecular mechanism through which lncRNAs modulate CRC development and progression remains largely unknown.

In this study, we found that many lncRNAs were differentially expressed in CRC tissues and normal tissues. Among them, SLCO4A1-AS1 was the most upregulated lncRNA. Evidence from TCGA database also showed that SLCO4A1-AS1 possesses a high frequency of copy number amplification in various cancers, especially in CRC. In addition, the overexpression of SLCO4A1-AS1 is linked to poor prognosis and tumor malignance in CRC. Knockdown of SLCO4A1-AS1 significantly inhibited the proliferation, migration and invasion of CRC cells and induced apoptosis in vitro and in vivo. Mechanistically, SLCO4A1-AS1 remarkably activates Wnt/β-catenin signaling pathway. SLCO4A1-AS1 enhanced the stability of β-catenin by inhibiting GSKβ-mediated phosphorylation. Altogether, we demonstrated that SLCO4A1-AS1 is an oncogene in CRC by activation of the β-catenin signaling.

## Methods

### Human samples

Human colorectal samples were collected from the First Affiliated Hospital of Harbin Medical University. And the clinicopathological features were listed in Table 1. These samples were divided into SLCO4A1-AS1 high or low group (the median value of SLCO4A1-AS1 expression as the cutoff), followed by survival rate analysis. Informed consent allowing use of these samples was obtained from each patient. Samples were processed according to the standard procedures with appropriate ethical approval by the Ethics Committee of the First Affiliated Hospital of Harbin Medical University.

**Table 1:** Association between SLCO4A1-AS1 expression and clinicopathological characteristics in 50 patients with CRC

Characteristics	SLCO4A1-AS1 Low	High^a^	Chi-square	*P* value
All cases	21	29		
Age (year)			1.643	0.200
< 60	16	26		
≥60	5	3		
Gender			0.006	0.939
Male	15	21		
Female	6	8		
Size (cm)			4.711	0.030
< 3	13	9		
≥3	8	20		
Lymph node metastasis			4.217	0.040
No	11	7		
Yes	10	22		
TNM			4.433	0.035
I/II	12	8		
III/IV	9	21		

^a^The median expression level was chosen for cutoff

For analysis of correlation between SLCO4A1-AS1 levels and clinical features, Pearson’s chi-square tests were used. Results were considered statistically significant at *P* < 0 .05

### Cell lines and cell culture

Human normal colorectal mucosa cell FHC and CRC cell lines (HCT116, HCT8, HT29, SW480, LOVO and SW620) were purchased from the American Type Culture Collection (ATCRC) and maintained according to the standard procedures.

### Cell transfection

shRNAs (shSLCO4A1-AS1: 5’-GCCTGAGCTTGTTCACAAA-3′) were designed using Clontech RNAi Target Sequence Selector and constructed into the pSiCoR plasmid according to the instructions. Two hundred ninety-three T cells transfected with pSiCoR as well as VSVG, RRE and RSV-REV were used to generate virus. HCT116 and SW480 cells were infected with virus supernatants. Stable cell lines were isolated by GFP sorting.

### Antibodies

Antibodies for Vimentin (1:2000; #5741, Cell Signaling Technology), E-cadherin (1:1000; #14472, Cell Signaling Technology), AXIN2 (1:2000; #5863, Cell Signaling Technology), MYC (1:5000; #13987, Cell Signaling Technology), β-catenin (1:1000; #8480, Cell Signaling Technology), phospho-β-catenin (T41, S45) (1:1000; #9565, Cell Signaling Technology), GSK3β (1:1000; #12456, Cell Signaling Technology) and GAPDH (1:5000; #5014, Cell Signaling Technology) were bought from Cell Signaling Technology. α-catenin (1:2000; #610194, BD) was from BD Transduction Laboratories. Fibronectin (1:1000; SAB4500974, Sigma) and LGR5 (1:1000; SAB2700211, Sigma) were from Sigma.

### Apoptosis analysis

Cell apoptosis were analyzed by flow cytometry (FACScan; BD Biosciences) using CellQuest software (BD Biosciences).

### Ubiquitination assay

This assay was carried out as described before [24]. In brief, HA-ubiquitin vector were transfected into CRC cells. Forty-eight h after transfection, cells were treated with MG132 (10 μM/L) for 10 h. Then cell lysates were pulled down using anti-β-catenin or IgG. Eluates were separated by SDS-PAGE and immunoblotted with anti-K48-Ub.

### Tumorigenesis and metastasis assays in vivo

Animal experiments were s approved by the Medical Experimental Animal Care Commission at the First Affiliated Hospital of Harbin Medical University. To assess the effect in vivo of SLCO4A1-AS1 on tumorigenesis, shSLCO4A1-AS1 or control SW480 cells (2×10^6^ cells per mouse) were injected into the flanks of 5-week-old athymic nude BALB/c mice were manipulated nude recipients subcutaneously. Tumor volumes weights were determined at indicative time points. To evaluate the effects of SLCO4A1-AS1 on tumor metastasis in vivo, shSLCO4A1-AS1 or control SW480 cells (4×10^6^ cells per mouse) were injected into the spleen subcapsular of each BALB/c nude mice. Six weeks after injection, metastatic nodules in livers were counted under a dissecting microscope.

### Cell proliferation assays

CCK-8 and colony formation assays were used for evaluation of cell proliferation ability. For CCK-8 assay, cells were seeded into a 96-well plate. At indicative time points, 10 μl CCK-8 solution was added into each well and incubated for 2 h at 37 °C. Then, the absorbance at 450 nm was determined. For the colony formation assay, 2×10^3^ cells per well were seeded in a six-well plate and cultured for 2 weeks. Colonies were fixed with methanol and stained with 0.1% crystal violet (1 mg/ml).

### In vitro migration and invasion assay

For migration assay, 2×10^4^ cells were seeded in the top chamber (8-μm pore; BD Biosciences) with serum-free medium and the lower chamber was added with 10% fetal bovine serum medium. Cells were incubated for 24 h and then non-migrated cells were removed and the migrated cells on the lower side were fixed, stained with crystal violet and photographed with an IX71 inverted microscope (Olympus Corp., Tokyo, Japan). For invasion assay, 8×10^4^ cells were placed in the upper chamber coated with 100 μl Matrigel (BD Biosciences, MA). And other steps were the same as migration assay.

### Real-time quantitative PCR

TRIzol solution was used to extract total RNAs from sample tissues or cell lines according to the manufacturer’s protocol. The M-MLV reverse transcriptase (Promega) was used for cDNA synthesis. qRT-PCR was performed as previously described [25]. Gene expression was normalized to U6 or GAPDH and calculated according to the 2^−ΔΔCT^ method. Specific primer sequences were available if requested.

### In situ hybridization (ISH)

ISH were conducted as previously described [26]. The probe sequences for SLCO4A1-AS1 were as follows: 5′-GAAGCTAGATGCRCAGCTAAT-3′ and 5′-TTGCGTTCATCGGAACRCAGG-3′.

### RNA immunoprecipitation (RIP)

Cells were lysed with RIP buffer (20 mM Tris pH 7.5, 150 mM NaCl, 1 mM MgCl2, 0.1% NP40, 5% glycerol and 0.5 mM DTT) supplemented with RNase inhibitor, followed by addition of specific antibody. RNA–protein complexes were enriched by Protein A/G beads. Then precipitated RNAs were eluted and used for cDNA synthesis.

### RNA pulldown

Biotin-labeled RNAs were obtained by the MaxiScript T7 kit (Ambion) with biotinylated CTP. Biotin-labeled RNAs in refolding buffer (10 mM Tris pH 7.5, 0.1 M KCl and10 mM MgCl2) were added into cell lysates and incubated for 4 h at 4 °C, followed by addition of beads. After washed 4 times, beads were boiled and precipitated proteins were checked with western blot.

### Statistical analysis

SPSS version 20.0 software (SPSS Inc., Chicago, IL, USA) was used for statistical analysis. Data was expressed as mean ± SD. Survival curves were calculated using the Kaplan-Meier curve followed by log-rank test. One-way ANOVA analysis or two-tailed Student’s t-tests were performed for *p*-value analysis, as appropriate. *P* < 0.05 was considered statistically significant.

## Results

### SLCO4A1-AS1 is overexpressed in human CRC

**Fig. 1:**
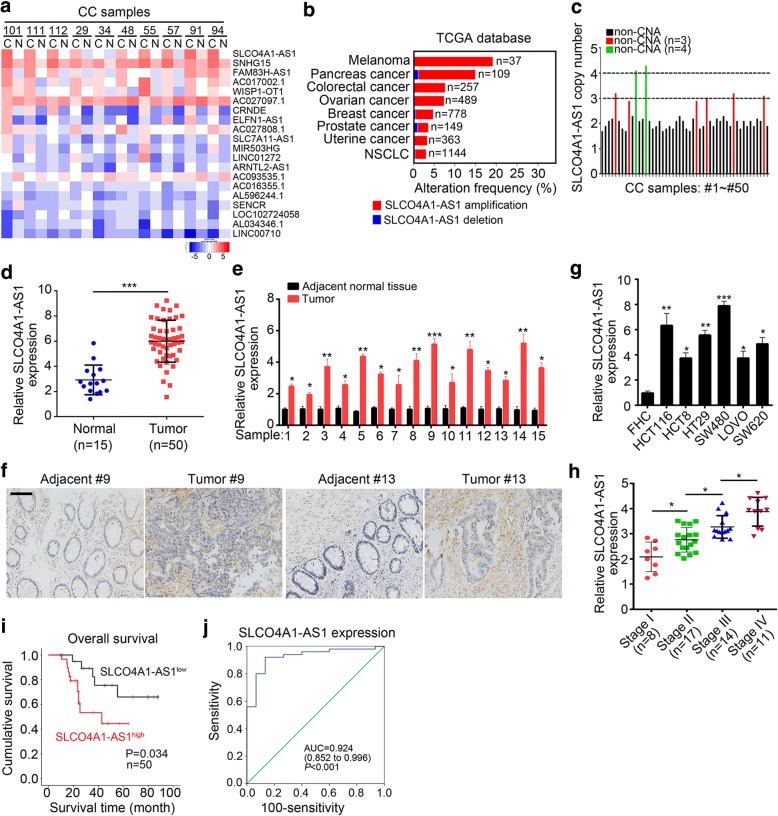
SLCO4A1-AS1 is overexpressed in human CRC*.* **a** Heatmap of differentially expressed long noncoding RNAs in CRC tissues and adjacent non-tumor tissues according to an online microarray dataset (GSE104836). **b** The copy number amplifications (CAN) of SLCO4A1-AS1 were determined according to TCGA database. **c** The CNA of SLCO4A1-AS1 was measured by qRT-PCR in 50 CRC samples. DNAs were extracted from samples and specific primers targeting SLCO4A1-AS1 DNA were used. ACTB DNA was used for loading control. **d** SLCO4A1-AS1 expression was determined by qRT-PCR in CRC samples (*n* = 50) and adjacent normal tissues (*n* = 15). **e** SLCO4A1-AS1 expression was examined by qRT-PCR in 15 pairs of CRC samples and adjacent normal tissues. **f** The expression of SLCO4A1-AS1 was determined by in situ hybridization in pairs of CRC samples and adjacent normal tissues (#9 and #13). Scale bar, 50 μm. **g** The expression levels of SLCO4A1-AS1 were determined by qRT-PCR in human normal colorectal mucosa cell FHC and CRC cell lines (HCT116, HCT8, HT29, SW480, LOVO and SW620). **h** 50 CRC samples were divided into four groups based on clinical stages (TNMs). And the expression of SLCO4A1-AS1 was measured in each group by qRT-PCR. **i** The effect of SLCO4A1-AS1 expression on clinical prognosis was determined by Kaplan–Meier survival analysis. Median value was the cutoff threshold of Kaplan–Meier survival analysis. **j** Prediction of CRC based on SLCO4A1-AS1 expression was analyzed by ROC curve using adjacent normal tissues as control. *p < 0.05, **p < 0.01 and ***p < 0.01

To identify CRC-related lncRNAs, we analyzed an online non-coding RNA profiling according to Li’s cohort (GSE104836) consisting of 10 pairs of tumor and adjacent normal tissues. Among all differentially expressed lncRNAs between tumor and normal tissues, SLCO4A1-AS1 was the most upregulated in tumor samples (Fig. 1a). Previous study showed that copy number amplification (CNA) is linked to upregulation of oncogene expression and subsequent tumorigenesis [27]. According to TCGA database, we found that the frequency of CNA of SLCO4A1-AS1 was very high in various cancers, including about 9% in CRC (Fig. 1b). To validate it, we collected 50 CRC samples and detected the copy number of SLCO4A1-AS1 by qPCR. We found that 8 samples had copy number amplification including 6 3-copy and 2 4-copy samples (Fig. 1c), which implied that SLCO4A1-AS1 may be involved in CRC development. To further validate it, we measured the expression of SLCO4A1-AS1 in CRC samples and adjacent normal tissues. We found that SLCO4A1-AS1 was significantly upregulated in CRC tissues compared with paired normal tissues (Fig. 1d and e). ISH assay also showed that the expression of SLCO4A1-AS1 was higher in CRC samples (Fig. 1f). Consistently, the expression of SLCO4A1-AS1 was higher in CRC cell lines (HCT116, HCT8, HT29, SW480, LOVO and SW620) than that in human normal colorectal mucosa cell FHC (Fig. 1g). To further determine the relationship between SLCO4A1-AS1 expression and tumor malignance, we checked the expression of SLCO4A1-AS1 in different stages of CRC samples and found that SLCO4A1-AS1 expression is positively correlated with clinical grade (Fig. 1h). Furthermore, by Kaplan–Meier survival analysis, we found that CRC patients with higher expression of SLCO4A1-AS1 showed poorer prognosis (Fig. 1i). In addition, receiver operating characteristic (ROC) curve was performed to evaluate the sensitivity and specificity of SLCO4A1-AS1 expression in predicting CRC tissues from normal tissues. The area under curve (AUC) was 0.924, which indicated SLCO4A1-AS1 might be a good predictor in CRC (Fig. 1j). Collectively, above results implied that SLCO4A1-AS1 was significantly upregulated and might serve as a biomarker for prognosis in CRC.

### SLCO4A1-AS1 knockdown inhibits cell proliferation, migration and invasion in CRC

**Fig. 2:**
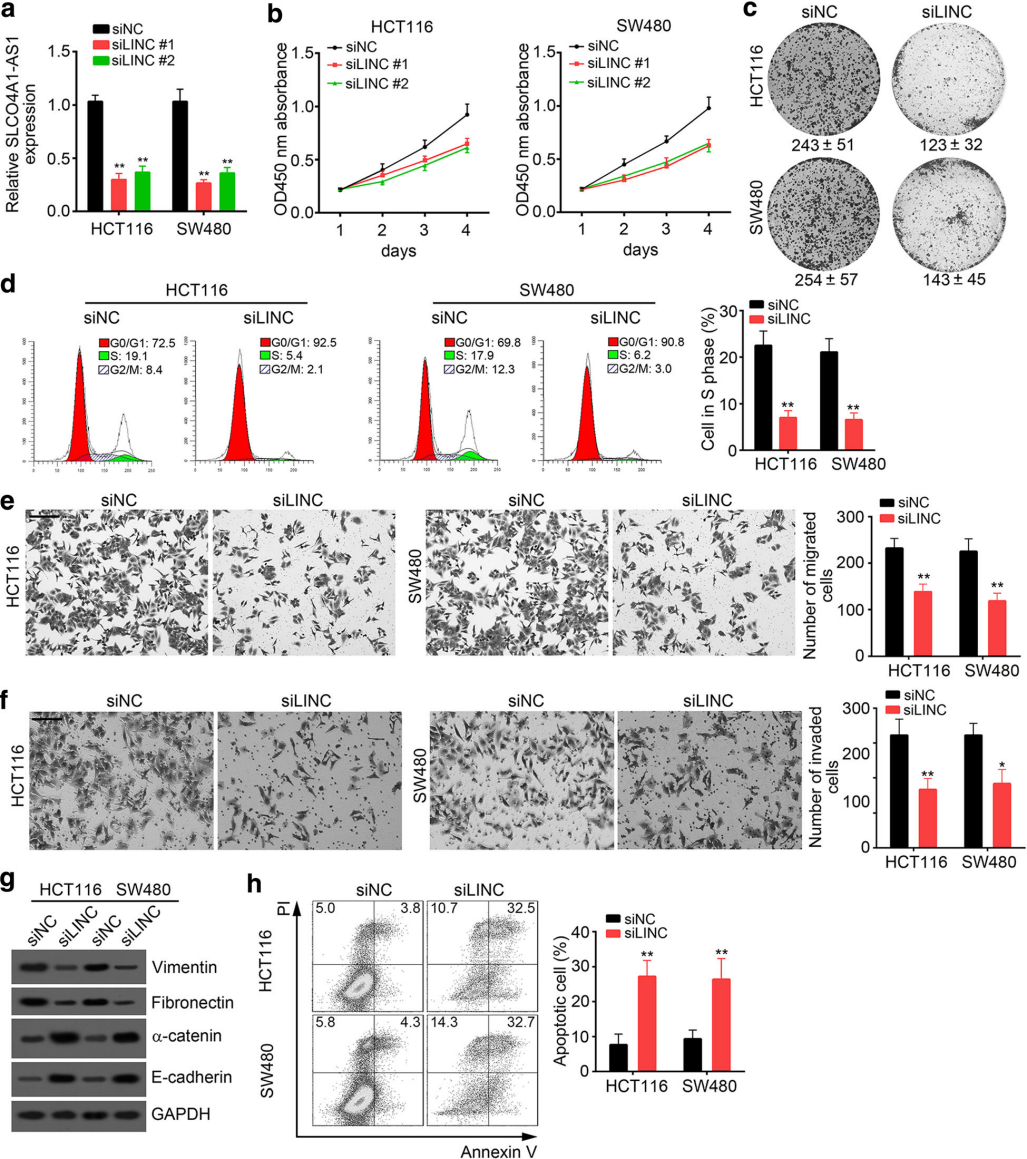
SLCO4A1-AS1 knockdown inhibits cell proliferation, migration and invasion in CRC. **a** Stable knockdown of SLCO4A1-AS1 was checked by qRT-PCR in HCT116 and SW480 cells transfected with lentivirus containing SLCO4A1-AS1 shRNA sequence. siNC: shRNA negative control; siLINC: shSLCO4A1-AS1. **b, c** The effect of SLCO4A1-AS1 expression on cell proliferation was evaluated with HCT116 and SW480 cells by CCK-8 assays and colony formation assays. **d** Cell cycle distribution was determined by FACS. **e, f** The effect of SLCO4A1-AS1 knockdown on the migration and invasion of CRC cells was assessed by a Transwell and Matrigel assay, respectively. Results are representative of the average counts from five random microscopic fields. Scale bar, 50 μm. **g** SLCO4A1-AS1 knockdown increased the expression of epithelial markers of α-catenin and E-cadherin while downregulated that of mesenchymal markers of Fibronectin and Vimentin in HCT116 and SW480 cells. **h** Knockdown of SLCO4A1-AS1 promoted the apoptosis of HCT116 and SW480 cells. Cells were stained with Annexin V/PI and analyzed by FACS. *p < 0.05, **p < 0.01 and ***p < 0.01

To investigate the role of SLCO4A1-AS1 in CRC, we knocked down SLCO4A1-AS1 with two independent siRNAs in HCT116 and SW480 cells (Fig. 2a). CCK-8 assays showed that SLCO4A1-AS1 knockdown led to growth retardation of HCT116 and SW480 cells (Fig. 2b). Colony formation assay similarly indicated that SLCO4A1-AS1-depleted CRC cells formed fewer colonies than the controls (Fig. 2c). Predictably, SLCO4A1-AS1 significantly inhibited the percent of CRC cells in S phase (Fig. 2d). In addition, Transwell assays showed that SLCO4A1-AS1 silencing decreased CRC cell migration (Fig. 2e) and invasion (Fig. 2f). Besides, we performed western blot to evaluate whether SLCO4A1-AS1 regulates pithelial-mesenchymal transition (EMT) in CRC cells. Results demonstrated that knockdown of SLCO4A1-AS1 upregulated the expression of epithelial markers such as E-cadherin, α-catenin while decreased the expression of mesenchymal markers such as Vimentin and Fibronectin (Fig. 2g). Finally, we assessed the effect of SLCO4A1-AS1 on cell apoptosis. By staining with Annexin V/PI, we found that SLCO4A1-AS1 knockdown dramatically promoted the apoptosis (Fig. 2h). Taken together, our data demonstrated that SLCO4A1-AS1 knockdown could inhibit CRC proliferation and invasion in vitro.

### SLCO4A1-AS1 activates Wnt/β-catenin signaling in CRC

**Fig. 3:**
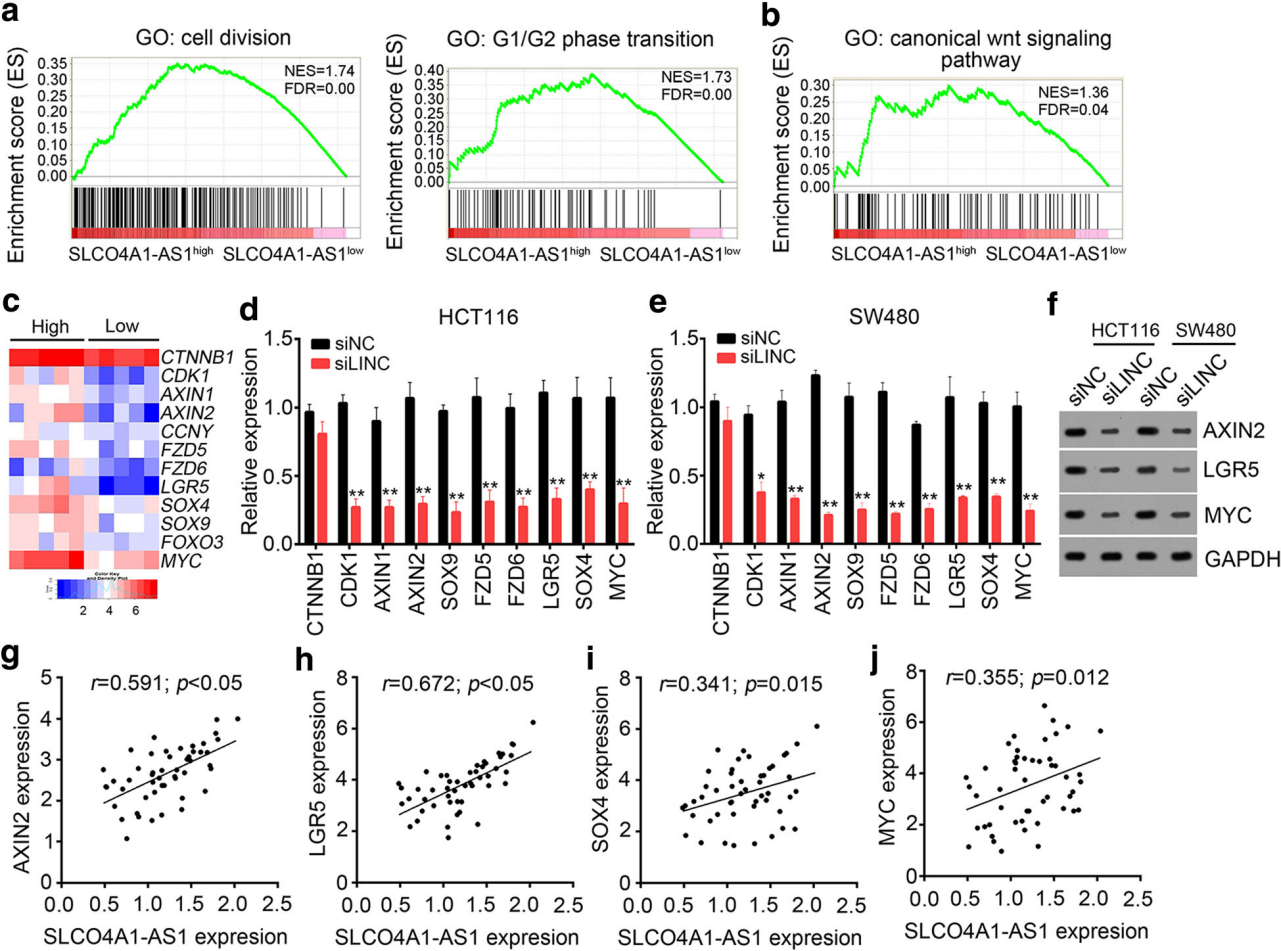
SLCO4A1-AS1 activates Wnt/β-catenin signaling in CRC. **a** Gene Set Enrichment Analysis (GSEA) showed that SLCO4A1-AS1 highly expression was positively related to cell division and G1/G2 phase transition. Samples in Li’s cohort (GSE104836) were divided into 2 groups based on expression of SLCO4A1-AS1. NES, normalized enrichment score. **b** GSEA showed that SLCO4A1-AS1 expression was positively correlated with canonical Wnt signaling according to Li’s cohort (GSE104836). **c** Expression heatmap of the target genes of Wnt/β-catenin signaling showed that more activation of Wnt/β-catenin signaling appeared in SLCO4A1-AS1^high^ group. **d, e** Real-time PCR confirmed that SLCO4A1-AS1 knockdown significantly downregulated Wnt/β-catenin signaling in HCT116 and SW480 cells. **f** The protein levels of AXIN2, LGR5 and MYC in HCT116 and SW480 cells after SLCO4A1-AS1 silence were measured by western blot (WB). **g-j** The mRNA levels of AXIN2, LGR5, SOX4 and MYC were positively correlated with the expression of SLCO4A1-AS1 in the 50 CRC samples. qRT-PCR was performed and *GAPDH* acted as loading control. *p < 0.05 and **p < 0.01

To further determine SLCO4A1-AS1-mediated molecular mechanism, we performed analysis in bioinformatics according to the Li’s cohort (GSE104836). We divided the 10 CRC samples in Li’s cohort into two groups based on SLCO4A1-AS1 expression (median value as the cutoff). Gene Set Enrichment Analysis (GSEA) showed that SLCO4A1-AS1 highly expression was positively related to cell division and G1/G2 phase transition (Fig. 3a), which supported our above results that SLCO4A1-AS1 promoted CRC cell proliferation. Some signaling pathways such as Wnt/β-catenin signal and NOTCH signal were reported to be involved in human cancers [28, 29]. We analyzed the relationship of SLCO4A1-AS1 with cancer-related signaling pathways and found that SLCO4A1-AS1 expression was positively correlated with Wnt/β-catenin signaling pathway (Fig. 3b). As shown, many target genes of Wnt/β-catenin signaling was downregulated in SLCO4A1-AS1^low^ CRC samples compared with SLCO4A1-AS1^high^ samples (Fig. 3c). Similarly, SLCO4A1-AS1 knockdown significantly decreased the mRNA and protein levels of these target genes in HCT116 and SW480 cells (Fig. 3d-f). What’s more, the expression levels of AXIN2, LGR5, SOX4 and MYC were positively correlated with that of SLCO4A1-AS1 in the 50 CRC sample tissues (Fig. 3j), which indicated that SLCO4A1-AS1 regulates Wnt/β-catenin signaling in CRC.

### SLCO4A1-AS1 interacts with β-catenin

**Fig. 4:**
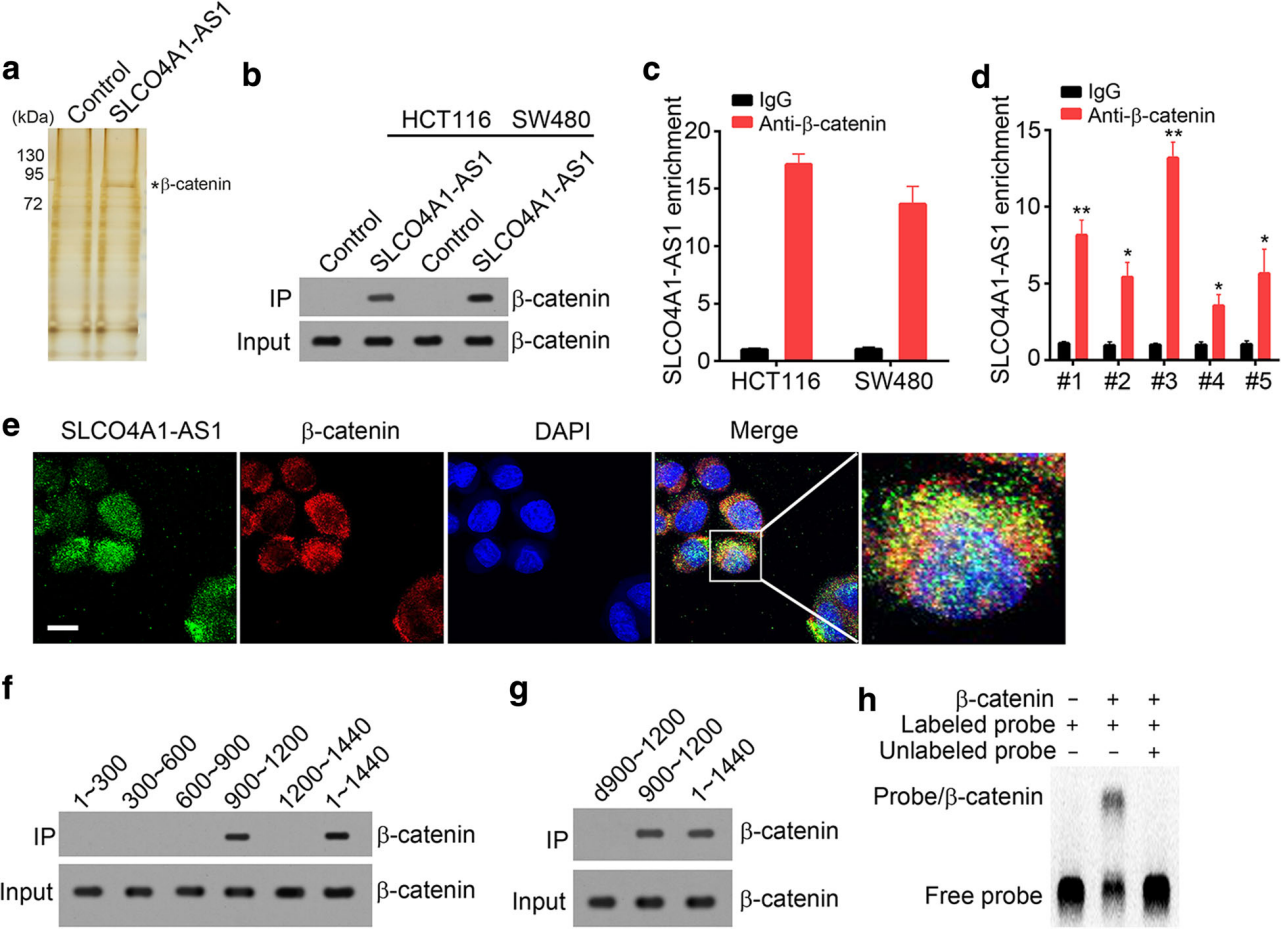
SLCO4A1-AS1 interacts with β-catenin. **a** RNA pulldown using biotin-labeled probe or intron control and sample lysates, followed by SDS-PAGE electrophoresis, silver staining and MS identification. **b** RNA pulldown showed that SLCO4A1-AS1 interacted with β-catenin in HCT116 and SW480 cells. SLCO4A1-AS1 was labeled with biotin. **c** RNA IP showed that β-catenin enriched SLCO4A1-AS1 in HCT116 and SW480 cell lysates. **d** β-catenin enriched SLCO4A1-AS1 in CRC sample cell lysates. **e** SLCO4A1-AS1 co-localized with β-catenin in CRC sample cells as shown by RNA fluorescence in situ hybridization (RNA-FISH). The scale bar was 10 μm. **f**, **g** Domain mapping showed that SLCO4A1-AS1 (nt: 900~ 1200) interacted with β-catenin and was indispensible. d900~ 1200 represented deletion of nt 900~ 1200. **h** RNA electrophoretic mobility shift assay (RNA-EMSA) showed that biotin-labeled SLCO4A1-AS1 (nt: 900~ 1200) directly bond to β-catenin. *p < 0.05 and **p < 0.01

We above showed that SLCO4A1-AS1 activated Wnt/β-catenin signaling in CRC. To determine how SLCO4A1-AS1 activates Wnt/β-catenin signaling, we performed RNA pulldown assays, followed by silver staining and mass spectrum (MS) identification. We identified β-catenin as an interactive protein of SLCO4A1-AS1 (Fig. 4a). To verify it, we performed pulldown assay and found that biotin-labeled SLCO4A1-AS1 precipitated endogenous β-catenin in HCT116 and SW480 cells (Fig. 4b). Furthermore, β-catenin antibody also enriched endogenous SLCO4A1-AS1 in HCT116, SW480 and CRC sample cells (Fig. 4c and d). In addition, SLCO4A1-AS1 co-localized with β-catenin in CRC sample cells (Fig. 4e). To further determine the essential region in SLCO4A1-AS1 for the interaction with β-catenin, we conducted domain mapping. We found that SLCO4A1-AS1 (nt 900~ 1200) directly bound to β-catenin (Fig. 4f). Moreover, deletion of this region (nt 900~ 1200) abrogated this interaction between SLCO4A1-AS1 and β-catenin (Fig. 4g). Furthermore, we performed RNA electrophoretic mobility shift assay (RNA-EMSA) with biotin-labeled probe (nt 900~ 1200) and demonstrated their direct association (Fig. 4h).

### SLCO4A1-AS1 increased the stability of β-catenin by inhibiting its phosphorylation

**Fig. 5:**
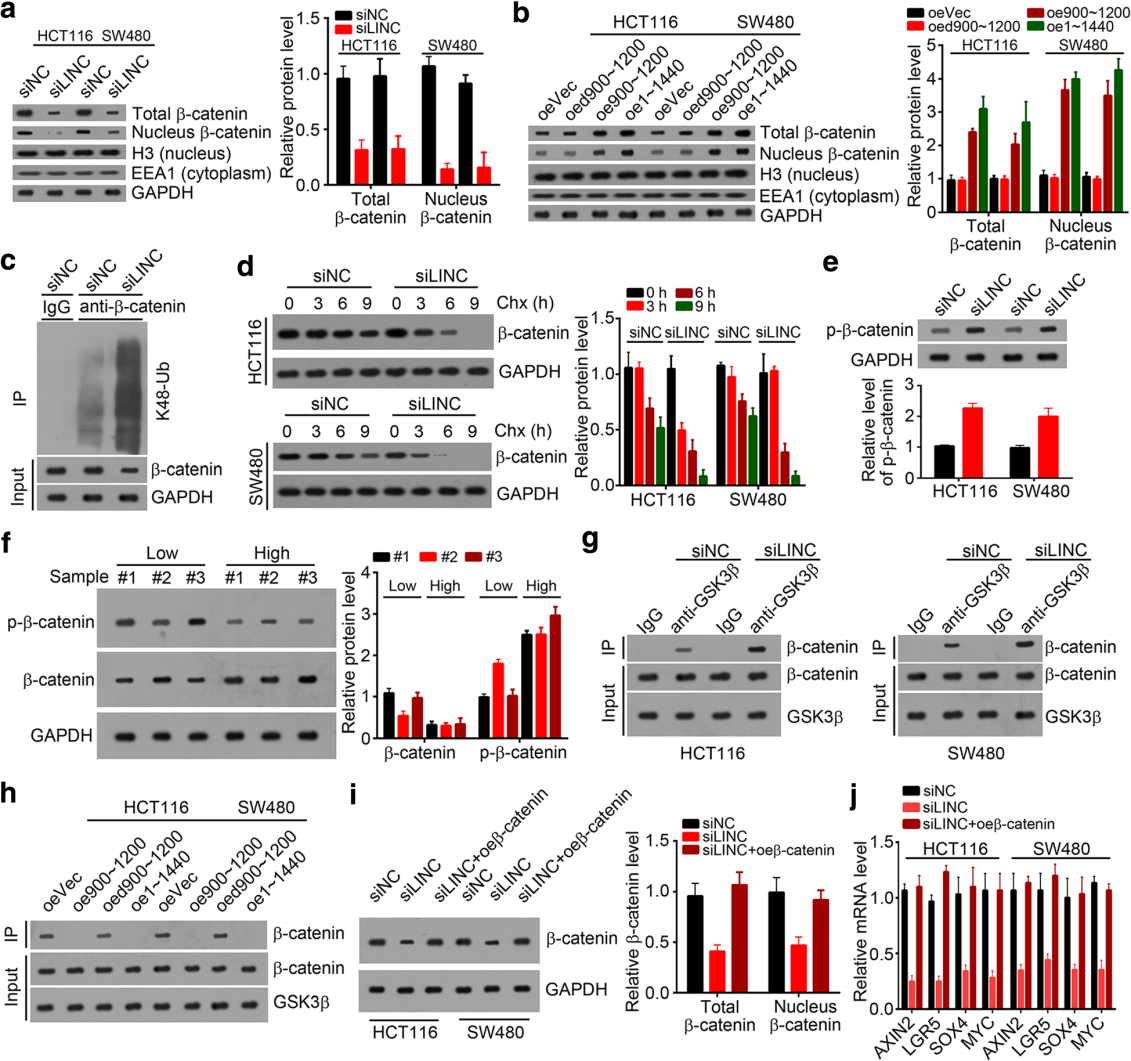
SLCO4A1-AS1 increased the stability of β-catenin by inhibiting its phosphorylation. **a** WB analysis showed that siRNA-induced SLCO4A1-AS1 knockdown decreased the protein level of β-catenin in HCT116 and SW480 cells. H3, nuclear marker; EEA1, cytoplasmic marker. **b** Overexpression of SLCO4A1-AS1 (full-length or nt 900~ 1200) promoted the protein level of β-catenin in HCT116 and SW480 cells. H3, nuclear marker; EEA1, cytoplasmic marker. **c** Knockdown of SLCO4A1-AS1 enhanced the ubiquitination of β-catenin in HCT116 cells. **d** SLCO4A1-AS1 knockdown accelerated the degradation of β-catenin in HCT116 and SW480 cells. Chx, cycloheximide. **e** SLCO4A1-AS1 knockdown promoted the phosphorylation of β-catenin in HCT116 and SW480 cells as shown by western blotting. **f** WB analysis showed that the protein levels of β-catenin were higher in SLCO4A1-AS1^high^ CRC samples while the phosphorylation of β-catenin was lower. **g** SLCO4A1-AS1 knockdown enhanced the interaction between β-catenin and GSK3β in HCT116 and SW480 cells. **h** WB analysis indicated that overexpression of SLCO4A1-AS1 (full-length or nt 900~ 1200) abrogated the interaction between β-catenin and GSK3β in HCT116 and SW480 cells. **i, j** Restoration of the protein levels of β-catenin by ectopic expression of β-catenin (**i**) rescued SLCO4A1-AS1 knockdown-induced inactivation of wnt/β-catenin signaling in HCT116 and SW480 cells (**j**)

We have confirmed the interaction between SLCO4A1-AS1 and β-catenin. Then we performed western blot and found that SLCO4A1-AS1 knockdown significantly decreased the protein level of β-catenin in HCT116 and SW480 cells (Fig. 5a). On the contrary, overexpression of full-lengthen or nt 900~ 1200 dramatically upregulated the protein level of β-catenin in HCT116 and SW480 cells (Fig. 5b). Additionally, we validated the elevated β-catenin ubiquitination signals using β-catenin immunoprecipitates from SLCO4A1-AS1–depleted HCT116 cells through (Fig. 5c) and consequently decreased β-catenin stability (Fig. 5d). A previous study showed that β-catenin phosphorylation by GSKβ promotes its ubiquitination-mediated degradation [30]. We then assessed the effect of SLCO4A1-AS1 on β-catenin phosphorylation and found that SLCO4A1-AS1 knockdown significantly increased β-catenin phosphorylation in HCT116 and SW480 cells (Fig. 5e). Moreover, SLCO4A1-AS1-overexpressed CRC sample tissues showed lower β-catenin phosphorylation (Fig. 5f). Besides, we found that SLCO4A1-AS1 knockdown enhanced the interaction between β-catenin and GSKβ in HCT116 and SW480 cells (Fig. 5g) while overexpressing SLCO4A1-AS1 abrogated their interaction (Fig. 5h). To further determine whether SLCO4A1-AS1 activated Wnt/β-catenin signaling by enhancing the stability of β-catenin, we restored the protein levels of β-catenin in HCT116 and SW480 cells (Fig. 5i). By qRT-PCR, we found that restoration of β-catenin rescued the activation of Wnt/β-catenin signaling in HCT116 and SW480 cells (Fig. 5j). Summarily, our results indicated that SLCO4A1-AS1 stabilized β-catenin by preventing the association between β-catenin and GSKβ, and consequently activated Wnt/β-catenin signaling in CRC.

### SLCO4A1-AS1 promotes CRC proliferation, migration and invasion by activating wnt/β-catenin signaling in vitro and in vivo

**Fig. 6:**
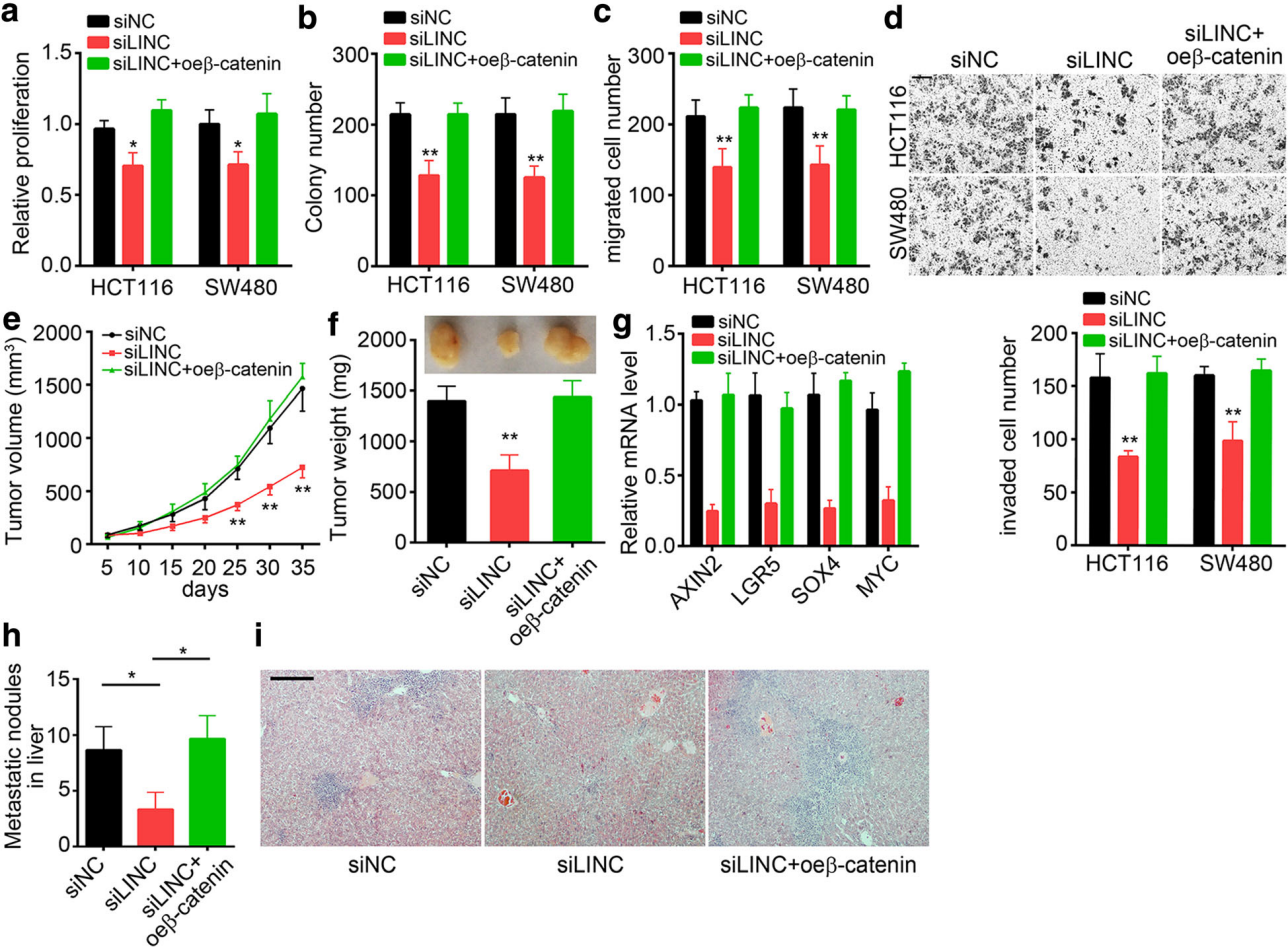
SLCO4A1-AS1 promotes CRC proliferation, migration and invasion by activating wnt/β-catenin signaling in vitro and in vivo. **a, b** Ectopic expression of β-catenin rescued the decreased proliferation ability of HCT116 and SW480 cells induced by SLCO4A1-AS1 knockdown as shown by CCK-8 and colony formation assays. **c, d** Overexpression of β-catenin restored the migration and invasion potential of HCT116 and SW480 cells as indicated by a Transwell and Matrigel assay, respectively. Scale bar, 50 μm. **e, f** SLCO4A1-AS1 knockdown delayed the tumor propagation in vivo while overexpressing β-catenin rescued it. Tumor volumes were measured at indicative time points. Tumor weights were measured at the end point of experiments. **g** Total RNAs were extracted from tumor tissues in **f** and the activation of wnt/β-catenin signaling was assessed by qRT-PCR. Results showed that SLCO4A1-AS1 knockdown downregulated wnt/β-catenin signaling while ectopic expression of β-catenin upregulated it in vivo. **h** Knockdown of SLCO4A1-AS1 expression remarkably reduced the metastatic nodules in the liver. **i** The liver sections were shown via H&E staining. Scale bar, 50 μm. *p < 0.05 and **p < 0.01

Whether the SLCO4A1-AS1-mediated augment of CRC cell growth and metastasis relied on activation of Wnt/β-catenin signaling was assessed in SLCO4A1-AS1-silenced HCT116 and SW480 cells transfected with β-catenin-overexpressing plasmid or empty control. Results showed that decreased proliferation, colony formation, migration and invasion potentials of SLCO4A1-AS1-silenced cells were rescued by ectopic expression of β-catenin in HCT116 and SW480 cells (Fig. 6a-d). What’s more, SLCO4A1-AS1 knockdown delayed tumor growth in vivo while overexpression of β-catenin in the meantime reversed it (Fig. 6e and f). Then we measured the activation of Wnt/β-catenin signaling in formed tumor tissues. As shown, the Wnt/β-catenin signaling was also downregulated in vivo after SLCO4A1-AS1 depletion (Fig. 6g). Finally, we evaluated the effect of SLCO4A1-AS1 on tumor metastasis in vivo, and found that SLCO4A1-AS1 knockdown severely reduced the metastatic nodules in the liver while β-catenin overexpression reversed this trend (Fig. 6h and i). Taken together, above data suggested that SLCO4A1-AS1 exerted functions dependent on activation of Wnt/β-catenin signaling in CRC.

## Discussion

In recent years, great efforts have been made to search cancer-related lncRNAs and determine their molecular mechanisms in tumor development and progression [31]. Here we identified the physiological functions of an uncharacterized lncRNA SLCO4A1-AS1 and determined its molecular mechanism. SLCO4A1-AS1 was highly expressed in CRC tissues and may act as a biomarker for CRC diagnosis. Notably, we detected an unbelievable high AUC, which might be due to the limited size of CRC samples. SLCO4A1-AS1 was found to promote the proliferation and invasion of CRC cells, indicating that it may be implicated in the process of tumorigenesis. Moreover, SLCO4A1-AS1 knockdown induced CRC cell apoptosis, which implied that SLCO4A1-AS1 may be important for the functional maintenance of normal cancer cells.

Further analysis showed that the activation of Wnt/β-catenin signaling was affected by SLCO4A1-AS1. As one of the most essential intracellular signaling pathways, Wnt/β-catenin signaling mediates diverse cellular processes, including embryonic development, cell proliferation, differentiation, migration, survival and so on [32–34]. Hyperactivation of the Wnt/β-catenin signaling often leads to various cancers such as liver cancer and CRC [35–37]. For instance, CRCAT-1-mediated activation of Wnt signaling pathway promotes cell proliferation and inhibits apoptosis in cervical cancer cells [38]. Additionally, activation of Wnt/β-catenin signaling by TGFβ promotes CRC development [39]. In our study, we found that SLCO4A1-AS1 knockdown severely decreased the protein level of β-catenin but not mRNA level by the mechanism that SLCO4A1-AS1 inhibited the phosphorylation and consequently ubiquitylation-mediated degradation of β-catenin. Through interacting with β-catenin, SLCO4A1-AS1 impaired the binding of GSK3β to β-catenin and inhibited β-catenin phosphorylation by GSK3β. Emerging evidence shows that lncRNAs can exert functions by regulation *in trans* [40]. lncRNAs may associate with proteins to regulate their stability, activity or other properties [11, 41, 42]. Based on above evidence, we proposed that SLCO4A1-AS1 may bind to β-catenin and then shield the interactive domain of β-catenin with GSK3β.

β-catenin level plays a pivot role in the canonical Wnt pathway [43]. Increase of β-catenin protein level may lead to abnormal cell proliferation and human diseases [44]. The regulation of β-catenin protein level is complicated and delicate. Phosphorylation and ubiquitylation of β-catenin are all reported to participate in the regulation of β-catenin stability [45]. For example, Liu et al. demonstrated that phosphorylation of β-catenin by CKIα in vivo is indispensible for subsequent phosphorylation of β-catenin by GSK3β, which finally leads to degradation of β-catenin [45]. Besides, other studies showed that phosphorylated β-catenin is ubiquitylated by E3 ubiquitin ligase β-TrCP and then degraded by the ubiquitin–proteasome pathway [46, 47]. Abrogation of β-catenin degradation promotes the accumulation of β-catenin in cells and induces tumor occurrence. For instance, inactivating mutation of APC, a pivot subunit of the degradation complex of β-catenin, gave rise to spontaneous CRC in mice [48]. So far, the regulatory mechanism of β-catenin turnover is not fully understood. Our study revealed that SLCO4A1-AS1 regulated the stability of β-catenin by weakening the association between β-catenin and GSK3β.

Continuous mutations of genes are popularly considered as a cause of tumors [49]. Gene copy number alterations or mutations are the common aberrances in cancers, and some studies have demonstrated the relevance between gene copy-number alterations and tumor formation and progression [50]. Previous study shows that DNA copy-number gain was observed on chromosome 20q in primary colorectal tumor [51]. Notably, SLCO4A1-AS1 is also located on chromosome 20q. Moreover, SLCO4A1-AS1 is really substantially amplified in CRC according to TCGA database and our experiment (Fig. 1b and c). However, how copy-number amplifications on chromosome 20q affect the expression and functions of SLCO4A1-AS1 in CRC remains further investigation.

## Conclusion

In summary, we found that lncRNA SLCO4A1-AS1 was highly expressed in CRC tissues. Upregulated SLCO4A1-AS1 promoted CRC progression through inhibiting the degradation of β-catenin by attenuating the interaction between β-catenin and GSK3β. This study revealed the vital significance of SLCO4A1-AS1 in CRC development.

## Abbreviations

CRC: colorectal cancer
EMSA: Electrophoretic mobility shift assay
lncRNA: long noncoding RNA
RNA-FISH: RNA fluorescence in situ hybridization
